# 

**Published:** 1838-03-14

**Authors:** 


### TO MEDICAL STUDENTS.

THE undersigned propose to receive a limited number of private pupils, from the first of April ensuing.

Their class will be examined carefully and repeatedly on all the various branches connected with medicine, and more particularly on the several courses of lectures which they may attend.

A COURSE on MINOR SURGERY will be delivered during the year, with all the incident operations. The student will be thus familiarized with the application of bandages, dressings of fractures and wounds; introduction of the catbether, the operations of blood-letting, tooth-drawing, vaccination, &c. &c. Abundant opportunities will be afforded him of frequently repeating these.

The various obstetric manipulations will be demonstrated on the machine. Each student will be enabled to practice these himself. Particular attention will be paid to the mechanism of labour.

The class will have access to a good library, including all the new Foreign and Domestic publications, Journals, &c.—also to a large and carefully selected Materia Medica collection.

Numerous preparations, models, &c. illustrative of the most difficult subjects of the different departments, will be placed at the use of the class.

M. CLYMER, M. D.
J. B. BIDDLE, M. D.

Office No. 230 Spruce street, between Sixth and Seventh sts. south side. March 14, 1838.

### TERMS.

The Examiner is published every alternate Wednesday, at *Three dollars* per annum, *payable in every instance in advance.* Two copies, if sent to the same address, for Five dollars.

Persons disposed to subscribe, will please send their address by mail, *accompanied with a remittance.*

Letters and communications may be addressed to the "Editors of the Medical Examiner," and must be *post paid, otherwise they will not be taken from the office.*

No subscriptions received for less than one year, commencing January 3d, 1838.

Advertisements inserted at the rate of $1 for twelve lines.

Office No. 230 Spruce street, between Sixth and Seventh sts. South side.

